# Global Trends in Hepatocellular Carcinoma and TGF-β Research: A Bibliometric and Visualization Analysis from 2000 to 2024

**DOI:** 10.2174/0113892037378714250529063227

**Published:** 2025-06-03

**Authors:** Liu-Lin Yang, Xing Chen, Kai-Ting Huang, Ji-Long Wang

**Affiliations:** 1 Department of Hepatobiliary Surgery, The First Affiliated Hospital of Guangxi Medical University, Nanning, China;; 2 Guangxi Key Laboratory of Enhanced Recovery After Surgery for Gastrointestinal Cancer, The First Affiliated Hospital of Guangxi Medical University, Nanning, China;; 3 Department of Ultrasound, The First Affiliated Hospital of Guangxi Medical University, Nanning, China

**Keywords:** Hepatocellular carcinoma, TGF-β, bibliometrics, visualization, hotspots, targeted therapies

## Abstract

**Background:**

Transforming growth factor-beta (TGF-β) plays a pivotal role in advanced hepatocellular carcinoma (HCC) by modulating immune responses, inflammatory processes, and epithelial-mesenchymal transition (EMT) in hepatocytes. It has emerged as a key therapeutic target for HCC.

**Objective:**

This study employs bibliometric analysis to examine literature published between 2000 and 2024, aiming to explore the critical roles of TGF-β in HCC and provide a theoretical foundation for future research.

**Methods:**

This study utilized the Web of Science Core Collection (WoSCC) database to analyze publications from January 1, 2000, to October 16, 2024. Visualization tools such as CiteSpace, VOSviewer, and SCImago Graphica were utilized to assess publication trends, countries, institutions, journals, authors, keywords, and references, identifying hotspots, trends, and the evolution of TGF-β research in the context of HCC.

**Results:**

The analysis encompassed 3,026 publications originating from 79 different countries. China was identified as the leading country in publication volume, with Fudan University being the most prolific institution. The journal Hepatology stood out as the leading publication in terms of both the volume of articles and citation influence. Keyword analysis revealed that recent research (2020–2024) has focused on metabolic regulation, the tumor immune microenvironment, and targeted therapies related to the TGF-β signaling pathway in HCC.

**Conclusion:**

This study highlights the publication landscape, research trends, and hotspots of TGF-β-related HCC research from 2000 to 2024, providing valuable insights and a theoretical basis for future studies in this critical field.

## 1 INTRODUCTION

Liver cancer poses a significant global health challenge due to its steadily increasing incidence rate, projected to affect over one million individuals by 2025, with hepatocellular carcinoma (HCC) accounting for approximately 90% of cases. Due to its frequent diagnosis at advanced stages, HCC is characterized by high postoperative recurrence and metastasis rates, resulting in poor patient outcomes. Studies have shown that the development of HCC is closely associated with chronic inflammation, viral infections (*e.g.,* hepatitis B and C), metabolic disorders, and toxin exposure [1-3]. Fur-thermore, alcohol consumption is widely recognized as a significant risk factor for HCC. Studies have shown that even moderate alcohol intake can increase the risk of liver cancer, particularly in individuals with chronic liver diseases such as cirrhosis. Alcohol promotes oxidative stress, inflammatory responses, and hepatocyte damage, which exacerbate liver fibrosis and facilitate the development of liver cancer [4-6]. Therefore, controlling alcohol consumption plays a critical role in the prevention of liver cancer. HCC is an extremely deadly cancer, especially in advanced stages, with a mortality rate reaching around 40%. Currently, Sorafenib continues to be the sole medication authorized by the U.S. Food and Drug Administration for managing advanced HCC [7]. More recently, other therapeutic agents such as lenvatinib, regorafenib, cabozantinib, and the VEGFR2 antagonist ramucirumab have also gained approval for the treatment of HCC, but the median survival of patients remains less than 15 months [8-11]. Although immune checkpoint inhibitors (such as nivolumab, pembrolizumab and astemizumab) have shown efficacy in certain patients, the tumor suppression rate remains low, ranging from 14% to 17% [12-15]. Therefore, there is an urgent need for more effective immunotherapeutic strategies and the identification of novel molecular targets.

Transforming growth factor-beta (TGF-β) is a versatile family of cytokines that plays a crucial role in numerous biological functions, such as regulating cell growth, differentiation, migration, immune responses, fibrosis, and the development of tumors [16, 17]. The role of TGF-β in HCC is complex and dualistic, acting as a tumor suppressor in the early stages of HCC and as a promoter of malignant transformation during HCC progression. In hepatocytes, TGF-β initially inhibits tumorigenesis by inducing cell cycle arrest and apoptosis [18, 19]. However, as tumors progress, TGF-β signaling shifts to promote tumor growth, migration, and invasion, primarily by activating PDGF or EGF signaling pathways, facilitating cell proliferation and anti-apoptotic mechanisms [20]. Additionally, TGF-β promotes epithelial-mesenchymal transition (EMT) by reducing the expression of epithelial markers like E-cadherin and increasing mesenchymal markers such as vimentin, ultimately facilitating tumor cell migration and invasion. This mechanism is strongly interconnected with various other signaling pathways, such as the upregulation of SNAI-1, which further enhances anti-apoptotic signals [21, 22]. Moreover, TGF-β inhibits apoptosis *via* the PI3K/AKT pathway and activates enzymes such as TACE/ADAM17, leading to the transactivation of EGFR signaling, which supports HCC cell proliferation and survival [23-25]. Within the tumor microenvironment, TGF-β modulates the behavior of HCC-associated fibroblasts, stimulating the release of growth factors and cytokines that drive tumor growth, invasion, and the formation of new blood vessels [26]. The genetic profile of TGF-β in HCC exhibits significant variations, with late-stage TGF-β signatures often associated with tumor recurrence and more aggressive phenotypes [27]. Furthermore, TGF-β enhances the stemness and malignant progression of HCC cells by inducing EMT and regulating stemness gene expression. PEG10, a key protein driven by TGF-β during EMT, is strongly linked to unfavorable prognosis and the recurrence of tumors in HCC [28, 29]. Consequently, TGF-β serves a crucial function in the clinical management and prognosis of HCC, with its dual functionality posing a complex therapeutic challenge.

Bibliometrics, through quantitative analysis of literature data, offers an effective approach to organizing and integrating a large body of scientific outputs. It reveals research trends and developmental dynamics within academic fields. In disease research, bibliometric analysis helps identify research hotspots, developmental directions, and key challenges related to specific diseases, providing researchers with cutting-edge academic insights and fostering innovation in emerging fields [30, 31]. To comprehensively understand the research progress on TGF-β signaling and HCC over the past 24 years, this study adopts a bibliometric visualization approach. It summarizes research outputs, growth trends, and citation frequencies. By constructing co-occurrence networks of countries/regions, institutions, and authors, as well as co-citation analysis of journals, the study highlights the key contributors to this field. Further analysis of keyword co-occurrence and emergent terms reveals research hotspots and frontiers, offering guidance for future studies. This integrative perspective enhances the understanding of the relationship between HCC and TGF-β, providing valuable references for the development of strategies to prevent and treat HCC.

## 2 MATERIALS AND METHODS

### 2.1 Data Collection and Screening

All relevant publications were sourced from the Web of Science Core Collection (WoSCC) database as of October 16, 2024. To guarantee the results are both reliable and accurate, the search terms were defined based on keywords and synonyms relevant to the research focus. The final search query was as follows: TS = (“hepatocellular carcinoma” OR “liver cell carcinoma” OR ” hepatic cell carcinoma” OR “primary liver carcinoma” OR “primary liver cancer” OR “malignant hepatoma” OR “hepatocarcinoma” OR “hepatoma”) AND TS = (“TGF-beta” OR “TGF-β” OR “TGFBETA” OR “Transforming growth factor-beta” OR “Transforming growth factor beta”). The search was limited to publications covering the period from January 1, 2000, to October 16, 2024, with document types restricted to research articles and reviews and the language set to English. A total of 3,176 documents were retrieved. After excluding 143 documents classified as meeting abstracts, proceeding papers, editorial materials, early access, retracted publications, book chapters, corrections, letters, retractions, and withdrawn publications, as well as 7 non-English articles, a final dataset of 3,026 records were obtained. This dataset includes complete information on the final selected publications and their cited references.

### 2.2 Data Filtering and Visualization Analysis

**Fig. (1):**
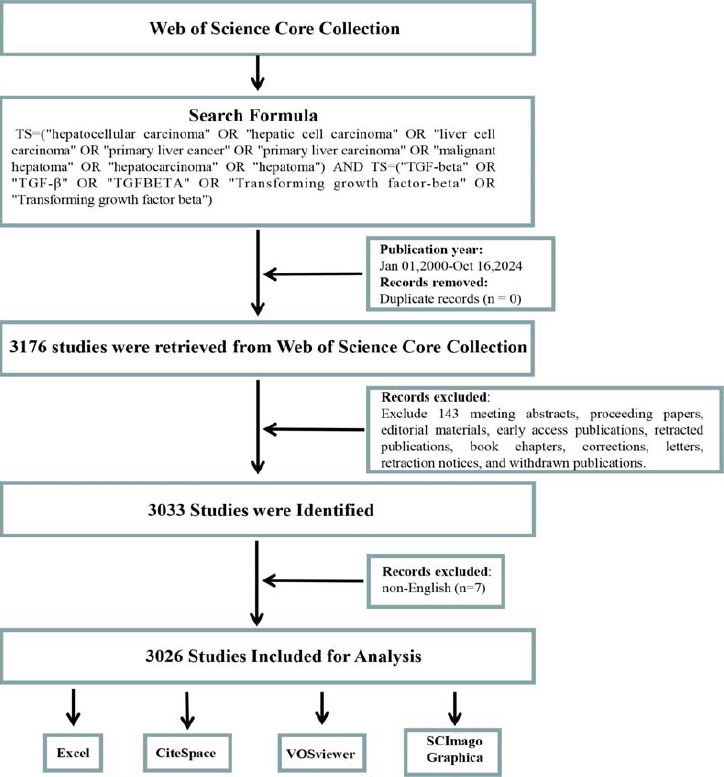
The overall workflow design for literature screening and visual analysis.

CiteSpace is a Java-based software tool developed for performing data analysis and generating visual representations [32]. It is widely used in bibliometrics to uncover research hotspots, emerging trends, and dynamic developments in academic fields [33]. Similarly, VOSviewer is an advanced software application designed for creating and visualizing bibliometric networks. It supports analyzing textual data and builds co-occurrence networks of critical terms in scientific literature. In this study, bibliometric data were processed utilizing CiteSpace (version 6.2.R3) and VOSviewer (version 1.6.20): VOSviewer was employed to generate network diagrams, analyzing publication volume and citation relationships among countries, institutions, journals, and authors. CiteSpace was used to produce burst detection maps of keywords and references, enabling the identification of sudden research hotspots within specific time periods. Additionally, Scimago Graphica (version 1.0.45) was utilized to create: World publication output maps showing geographic contributions to this field. Inter-country collaboration networks, visualizing the relationships between countries in this domain. Excel (2016) was used to create: Annual publication trends and cumulative publication growth graphs, illustrating changes in research output over time. A visual representation of the literature screening and analysis process used in this study is illustrated below (Fig. **1**). This combination of tools allowed for a comprehensive exploration of bibliometric characteristics, providing insights into research contributions, collaboration networks, and evolving themes within the TGF-β and HCC research field.

## 3 RESULTS

### 3.1 The Annual Publication Trend

**Fig. (2):**
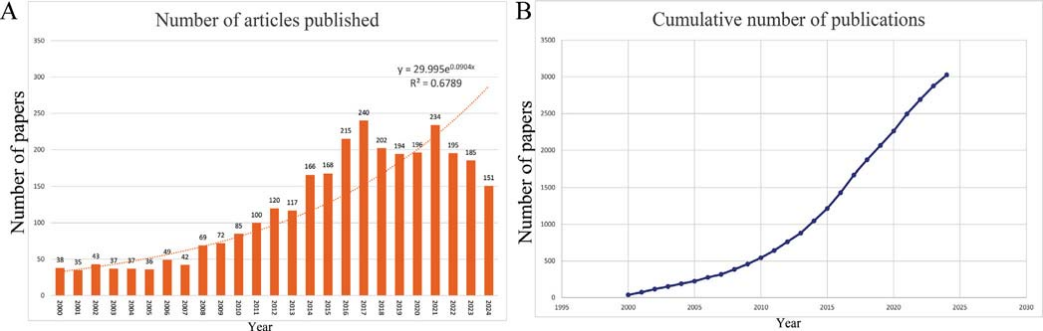
Charts in the growth of publication numbers on TGF-β and HCC. (**A**) Depicts the yearly publication counts and trends spanning from 2000 to 2024. (**B**) Shows the cumulative number of publications over the same period.

This study identified a total of 3,026 publications authored by 18,162 researchers from 79 countries, affiliated with 2,928 institutions, and published in 720 journals. From 2000 to 2005, an average of 37.7 articles on TGF-β and HCC were published annually, with a relatively stable trend. Between 2006 and 2017, the number of publications gradually increased, peaking in 2017 with 240 articles. From 2018 to 2020, research activity in this field slightly declined and plateaued at a stable level, but the overall output remained significantly higher than the pre-2017 levels. Between 2021 and 2024, the annual publication count showed a gradual decline (Fig. **2A**). The cumulative publication count graph reveals a distinct and steady rise in the number of studies conducted in this field over time (Fig. **2B**).

### 3.2 Analysis of Countries with the Highest Publication Outputs

**Fig. (3):**
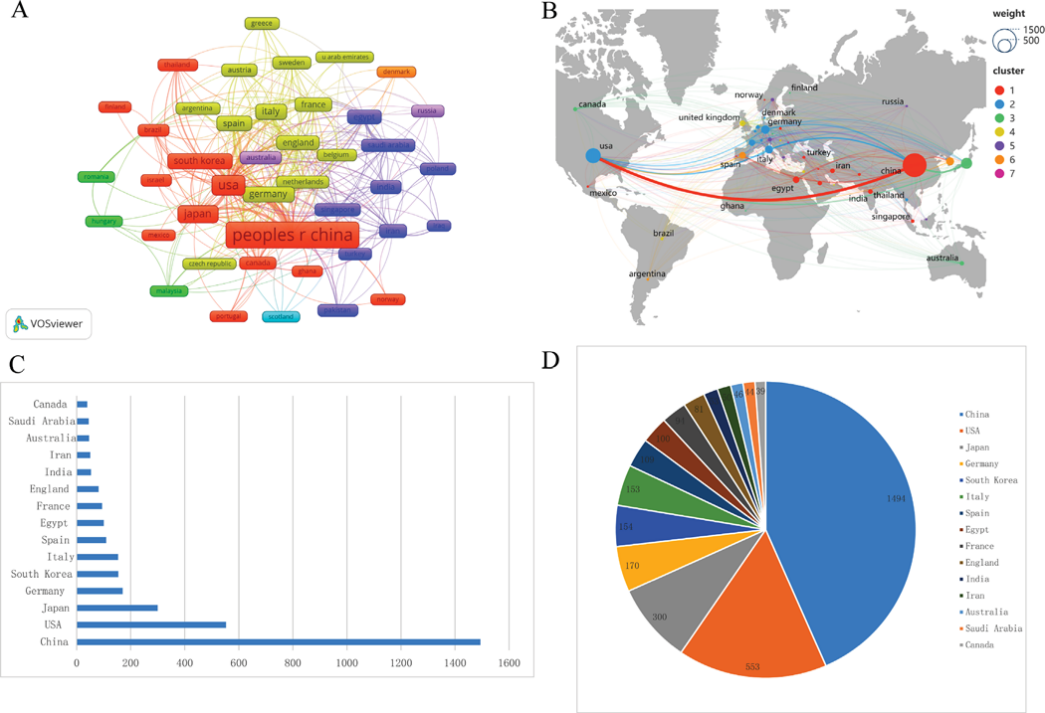
Worldwide research contribution. (**A**) Collaborative network map of countries generated using VOSviewer, displaying nations with a minimum of five publications. The size of the nodes represents the number of publications, while the color indicates the clustering of collaborative relationships. (**B**) A geographic distribution map was created using Scimago Graphica, visualizing the global publication output and international research collaborations in this field. (**C**) Bar chart illustrating the publication counts for each country. (**D**) Pie chart representing the percentage of publications contributed by each country.

Research on TGF-β and HCC spans 79 countries, with VOSviewer identifying 43 countries that published five or more articles to generate a collaborative network map (Fig. **3A**). Each node corresponds to a country, with its size reflecting the volume of publications, lines representing collaborative relationships between countries, and colors signifying clusters that reflect the strength of collaborations. Using SCImago Graphica, a corresponding world map was generated to visualize the geographic distribution of global scientific collaborations in this field. China is the largest node, leading in publication volume, followed by the United States. The thickest line connects China and the United States, indicating strong collaboration between the two nations. Research in this field is globally distributed without a significant clustering trend (Fig. **3B**). An in-depth analysis was conducted on the top 10 countries with the highest publication volumes. China ranked first with 1,494 publications, with the United States taking the second spot (553 papers), and Japan ranks third (300 papers). Germany (170 papers), South Korea (154 papers), Italy (153 papers), Spain (109 papers), Egypt (100 papers), France (94 papers), and England (81 papers) also contributed significantly. In terms of total citations, China led with 50,324 citations, followed by the United States (39,314 citations) and Japan (14,655 citations). Spain achieved the highest average citations per paper (82.64 citations), followed by England (79.72 citations) and the United States (71.09 citations) (Table **1**). Bar and pie charts provide a clearer visual representation of the top 15 countries' publication volumes in TGF-β and HCC research. Among these, developing countries include China, Egypt, India, Iran, and Saudi Arabia, while developed countries include the United States, Japan, Germany, South Korea, Italy, Spain, France, England, Australia, and Canada. China dominates the field, with a significantly higher publication output than other countries, highlighting its leading role in TGF-β and HCC research (Figs. **3C** and **D**).

### 3.3 Analysis of Institutions with the Highest Publication Outputs

**Fig. (4):**
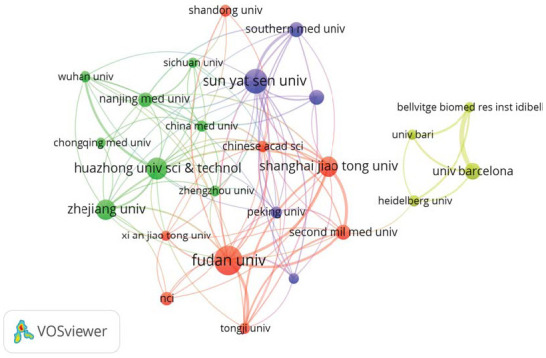
Institutional collaboration network based on publication volume generated using VOSviewer. The size of each node represents the number of publications contributed by the institution, while the connecting lines indicate the strength of collaborative relationships.

An interaction map of institutions with a minimum of 30 publications was created using VOSviewer (Fig. **4**). In this map, nodes represent research institutions, with node size indicating their impact and node color reflecting collaborative clusters. Fudan University, Sun Yat-sen University, Huazhong University of Science and Technology, and Shanghai Jiao Tong University dominate the collaboration network. Fudan University ranked first with 105 publications, followed by Sun Yat-sen University (84 papers) and Huazhong University of Science and Technology (73 papers). The University of Barcelona had the highest total citations (5,112 times), followed by the Naval Medical University of China (4,652 times) and Fudan University (4,114 times). The University of Barcelona recorded the highest average number of citations per publication (96.45 times). Among the top 10 institutions, 9 are based in China, while the remaining one is in Spain (Table **S1**). This demonstrates the leading role of Chinese institutions in TGF-β and HCC research with significant contributions.

### 3.4 Analysis of Top Publishing and Highly Co-cited Journals and Interrelations Between Citation and Co-citation

**Fig. (5):**
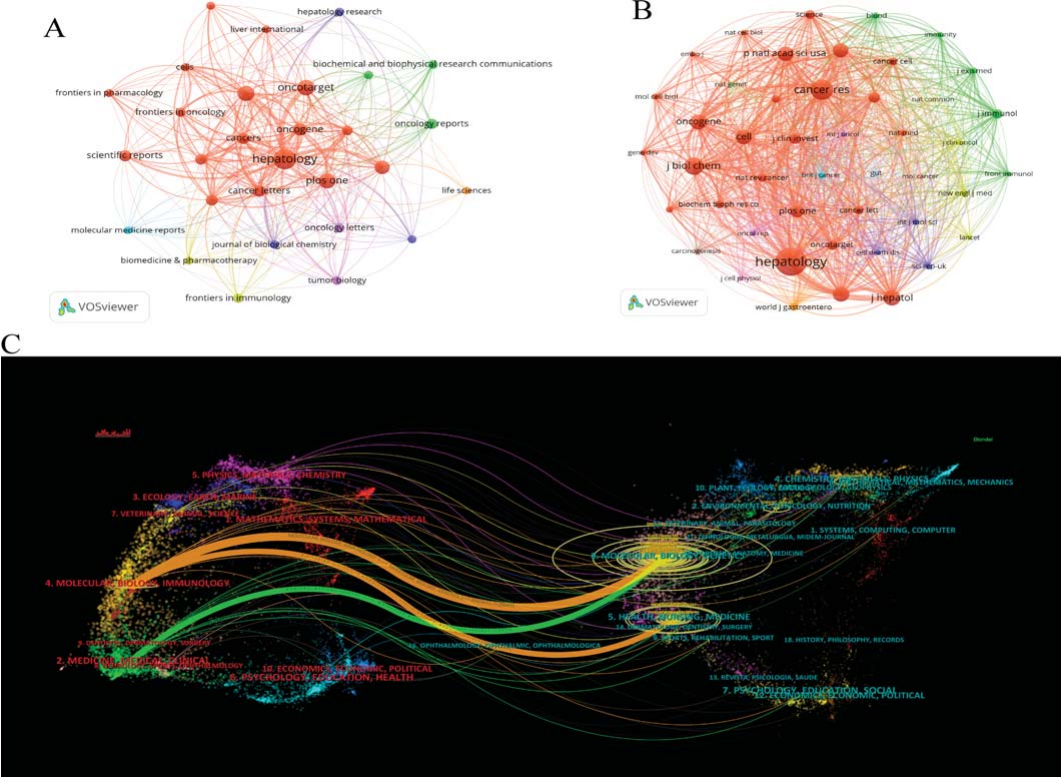
Visualization of Journals *via* VOSviewer and CiteSpace. (**A**) Journal interaction distribution map generated by VOSviewer, displaying journals with a minimum of 20 publications in this research field. (**B**) Network interaction map of journals with a minimum of 800 co-citations generated by VOSviewer. (**C**) Journal dual-map overlay visualizations produced using CiteSpace illustrate the citation and co-citation relationships between two groups with journals.

VOSviewer analysis revealed that, from 2000 to 2024, a total of 720 journals contributed to research on TGF-β and HCC, with 29 journals publishing no fewer than 20 related articles. Among these, Hepatology recorded the largest number of publications (106 papers), followed by Oncotarget (67 papers) and the International Journal of Molecular Sciences (66 papers). In terms of total citations, Hepatology ranked first with 11,920 citations, subsequently followed by Journal of Hepatology (4,302 citations) and Oncogene (4,140 citations). Moreover, the Journal of Hepatology had the highest average citations per article (119.5 times), followed by Hepatology (112.45 times) and Oncogene (90 times). Among the top 10 journals with the highest publication outputs, three were based in the United States, two each in Switzerland, the United Kingdom, and the Netherlands, and one in China (Table **S2**). The journal interaction network map highlights Hepatology as a central node, showing strong co-citation relationships with other journals such as Oncotarget and Cancer Letters, underscoring its significant influence in the field (Fig. **5A**).

Further analysis of co-cited journals revealed that Hepatology (9,059 co-citations), Cancer Research (5,740 co-citations), and Journal of Biological Chemistry (4,680 co-citations) were the most frequently co-cited journals (Table **S3**). Larger nodes in the network represent journals with higher citation counts and greater influence. Different colors in the co-citation network indicate distinct research focuses. Red nodes represent multidisciplinary journals related to oncology, molecular biology, and cell biology, such as Cancer Research, Oncogene, Cell, and PLOS ONE. Green nodes correspond to immunology-focused journals, including Journal of Immunology, Frontiers in Immunology, and Immunity, which emphasize the immune system, immune diseases, and immunotherapy. Purple nodes are associated with biochemistry and molecular biology journals, such as the Journal of Biological Chemistry and Biochemical and Biophysical Research Communications, focusing on biochemical processes, molecular interactions, and basic biological research (Fig. **5B**).

CiteSpace provided a dual-map overlay for more intuitive analysis of the citation and co-citation relationships in TGF-β and HCC research (Fig. **5C**). Each node represents a journal, with citation relationships depicted by colored curves from left to right, clearly presenting citation flow. The left panel (red font) represents citing journals, while the right panel (blue font) shows cited journals. Orange paths illustrates that journals in molecular immunology, cell biology, and genomics are frequently cited by journals in health, nursing, and pharmacology. Green paths indicate that literature in drug-clinical fields also influences molecular biology journals. This bidirectional interaction highlights the foundational role of basic research in clinical practice while emphasizing that new discoveries in drug development and clinical studies can, in turn, drive deeper exploration in molecular biology. This is particularly evident in studies of tumor molecular mechanisms, immune responses, and cellular interactions. Such interdisciplinary feedback and collaboration strengthen the connection between drug development and molecular biology research, further advancing precision medicine and personalized therapies. This virtuous cycle accelerates the translation of basic research into clinical applications, offering more precise and effective approaches for cancer treatment.

### 3.5 Analysis of Authors with the Highest Publication Outputs and Co-citations

**Fig. (6):**
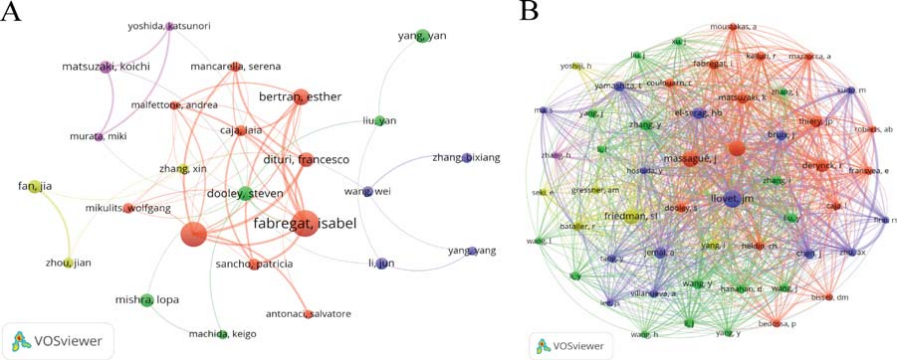
Author network visualization maps generated by VOSviewer. (**A**) Collaborative network analysis of authors who have published 10 or more research papers. (**B**) Co-citation network analysis of authors with a minimum of 120 co-citations.

Over the past 25 years, 18,162 authors have contributed to research on TGF-β and HCC. VOSviewer analysis was used to screen authors with at least 10 publications to generate a network interaction diagram (Fig. **6A**), and the top ten authors with the highest number of publications were selected and listed in a table (Table **2**). Among them, 29 authors published at least 10 papers. The authors with the most publications were Fabregat, Isabel (47 papers) from Spain and Giannelli, Gianluigi (43 papers) from Italy, who led other authors by a large margin, followed by Bertran, Esther (24) from Spain. The two authors with the most citations were also Fabregat, Isabel (3,241 times) and Giannelli, Gianluigi (3,239 times). Dooley, Steven, from Germany, had the highest average citation count (101.19 times) among the authors, followed by Mishra, Lopa from the United States (77.72 times), Italian author Giannelli, Gianluigi (75.33 times), and Spanish author Sancho, Patricia (73.2 times). Among the top ten authors in terms of number of publications, three are from Spain, two each from Italy and China, and one each from Germany, the United States, and Japan. Similarly, authors with at least 120 citations were selected to generate an interaction graph (Fig. **6B**). Among the 72,359 co-cited authors, 54 authors had a co-citation threshold of more than 120 times. Co-cited authors are those who are frequently cited together across multiple publications. Co-citation analysis is a bibliometric method used to investigate the underlying knowledge framework and research hotspots in an academic research field [34]. The analysis found that the co-cited author, Llovet, JM, had the highest total number of citations (593 times), and Massagué, J, ranked second with 513 citations. Giannelli, G., who ranked third with 500 citations (Table **S4**).

### 3.6 Analysis of the Most Cited Documents and Most Frequently Occurring Keywords

**Fig. (7):**
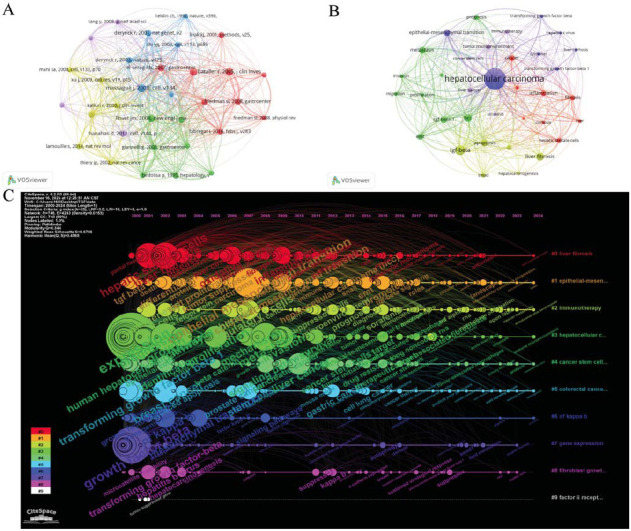
Visual representations of co-cited references and keywords created using VOSviewer. (**A**) Co-citation network of references cited 70 or more times. (**B**) Keyword co-occurrence network, displaying keywords that appeared at least 30 times. (**C**) A timeline view of the keywords in the article.

We used VOSviewer to construct an interaction network diagram (Fig. **7A**) of references with no less than 70 citations (32 articles in total). In the diagram, each node signifies a document, while the lines illustrate relationships between documents that are frequently co-cited in other literature. To enhance the citation analysis of references, we selected the ten most frequently cited works in this field from 2000 to 2024 for further examination (Table **S5**). The data showed that the paper with the most citations (174 times) was “TGF-β in Cancer,” published by Joan Massagué in Cell in 2008. The paper with the second highest citation count is “Liver Fibrosis,” published by Ramón Bataller in The Journal of Clinical Investigation in 2005, and “Global Cancer Statistics,” published by Ahmedin Jemal in CA: a cancer journal for clinicians in 2011. Both of these papers were cited 151 times.

In addition, the study screened out high-frequency keywords of key literature, and a total of 35 keywords with a frequency of ≥30 were chosen to produce a keyword co-occurrence network view (Fig. **7B**). From the perspective of the keyword co-occurrence network, in addition to the terms hepatocellular carcinoma and TGF-β as the central keywords themselves, liver fibrosis, metastasis, and apoptosis have the strongest correlation with the topic, indicating that TGF-β is involved in multiple molecular mechanisms such as liver fibrosis, tumor metastasis, and apoptosis in the pathogenesis of HCC. In addition, in order to enhance the clarity of keywords, we also used a table to display the number of occurrences of high-frequency keywords (Table **3**): liver fibrosis appeared 166 times, metastasis appeared 133 times, and apoptosis appeared 124 times.

Next, we clearly showed the research trends, hot spot evolution, and keyword associations of HCC and TGF-β signaling pathways from 2000 to 2024 (Fig. **7C**). The more connections between nodes, the stronger the correlation between keywords. Clusters of different colors represent different research topics: #0: Molecular mechanism of liver fibrosis, #1: Signal transduction of hepatocellular carcinoma, #2: Immunotherapy of hepatocellular carcinoma, #3: The key role of cancer stem cells and oxidative stress in the progression of hepatocellular carcinoma, #4: Transcriptional regulation of hepatocellular carcinoma, #5: Inhibition of liver cancer stem cells and synergistic regulation mechanism of anti-inflammatory factors, #6: Extracellular vesicles of hepatocellular carcinoma, #7: Growth factors and gene regulation of hepatocellular carcinoma, #8: Genetic variation and model research of hepatocellular carcinoma, #9: Insulin-like growth factor (IGF) signaling and angiogenesis in liver cancer. The nodes on each cluster represent the keywords of the topic, and the larger the node, the higher the keyword intensity. In addition, we also extracted the evolution of keywords in different time periods. We can observe that the keywords from 2000 to 2005 were mainly concentrated on keywords such as growth factor beta, receptor, growth factor, liver fibrosis, proteins, and tumor suppressor, reflecting the theme of basic disease mechanisms and protein receptor research; the keywords from 2005 to 2010 were concentrated on keywords such as therapy, hepatic fibrosis, mechanism, metastasis, invasion, survival, *etc.*, and the themes presented were related to the research on the mechanism of tumor invasion and metastasis and its treatment strategies; the keywords from 2011 to 2015 were mainly stem cells, microenvironment, prognosis, drug resistance, sorafenib, *etc.* The research focuses on deeper mechanism research, such as tumor stem cells, tumor microenvironment, and more attention to disease prognosis. At the same time, Sorafenib, classified as a tyrosine kinase inhibitor, is employed in the treatment of advanced HCC and has become a research hotspot. From 2016 to 2020, the keywords are concentrated on molecular mechanisms, long noncoding RNA, circular RNA, SAMD4, signaling pathway, *etc.*, and the research topics are concentrated on transcriptional regulation, molecular mechanisms, and signaling pathways. The shift in hot spots during this period reflects the in-depth exploration of the systematic and delicate mechanisms of gene expression regulation. From 2021 to 2024, the high-frequency keywords are lipid metabolism, targeted therapy, immune evasion, and immune microenvironment; the themes presented are reflected in metabolic regulation, tumor immune microenvironment, and its targeted therapeutic potential. From the evolution of keywords, we can conclude that the research on HCC and TGF-β has gradually extended from the basic single mechanism to the cellular level, signaling network, and tumor microenvironment and has made breakthroughs in the exploration of metabolic regulation and targeted immunotherapy. This evolution not only reflects the deepening and refinement of research but also reveals the future research direction: by integrating metabolism, immune regulation, and tumor microenvironment regulation, a multidimensional regulatory strategy is constructed, providing new possibilities for the precise treatment and clinical transformation of HCC.

### 3.7 Keyword and Reference Burst Analysis

**Fig. (8):**
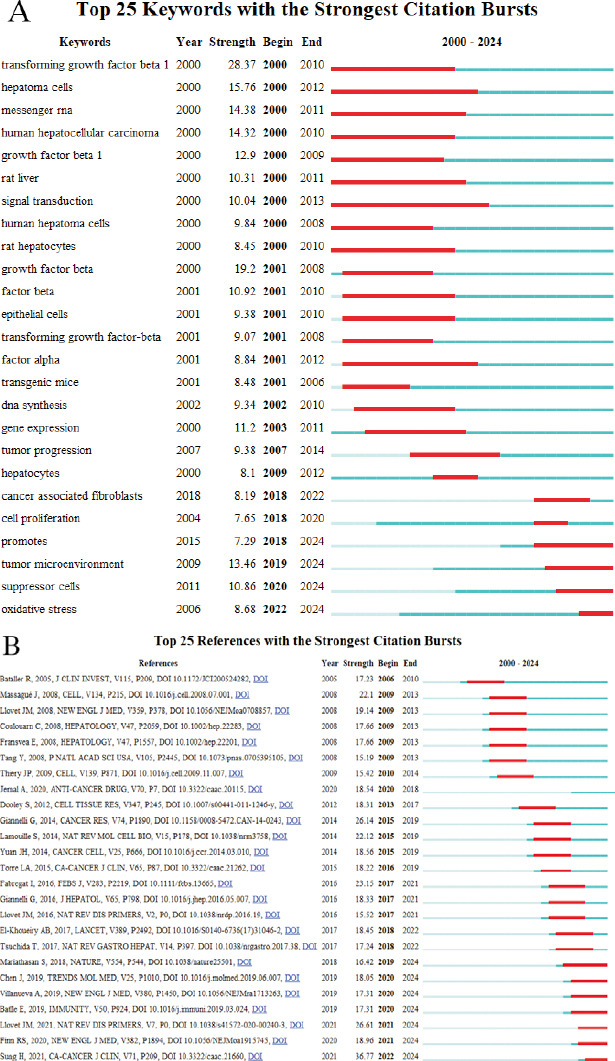
Burst map generated by CiteSpace. (**A**) Top 25 keywords with the strongest citation bursts. (**B**) Top 25 references with the strongest citation bursts.

We used Citespace to create a citation burst map of the 25 most powerful keywords in the field of TGF-β and HCC research from 2000 to 2024 (Fig. **8A**), uncovering the evolutionary trends of key research areas in this domain. From the figure, it is evident that the research hotspots have gone through three main stages: the early stage (2000-2010) was dominated by basic cytokine and cancer-related research, and the keywords that burst out during this period were “transforminggrowthfactorbeta1” (burst intensity 28.37), “hepatoma cells” (burst intensity 15.76) and “messenger rna” (burst intensity 14.38), *etc.*; the mid-term (2010-2018) turned to gene expression, phenotypic mechanisms, and tumor progression-related factors, and the burst keywords were “gene expression” (burst intensity 11.2) and “t In the near future (2019-2024), the focus will be on tumor microenvironment, tumor immune microenvironment, and oxidative stress, with the burst words “tumor microenvironment” (burst intensity 13.46), “suppressor cells” (burst intensity 10.86) and “oxidative stress” (burst intensity 8.68). In addition, we also made a reference burst map (Fig. **8B**), which contains detailed literature and author information, reflecting the references with high influence in different years, providing a sufficient theoretical basis for research in this field.

## 4 DISCUSSION

In the past 20 years, HCC research has made significant progress, and the importance of various cellular biological factors has attracted much attention from researchers. TGF-β is a cytokine with diverse biological functions, playing a key role in numerous processes, including cell growth, differentiation, migration, immune regulation, fibrosis, and tumorigenesis. TGF-β signaling pathway plays a complex dual role in the initiation and progression of HCC [35]. At the early stage, TGF-β inhibits the occurrence of HCC by inducing cell cycle arrest and promoting apoptosis. However, as the tumor progresses, the transformation of TGF-β signaling causes it to promote the proliferation, migration, and invasion of tumor cells in the tumor microenvironment [18, 19, 36]. Research has demonstrated that TGF-β promotes the survival and growth of tumor cells through the activation of various signaling pathways, such as PDGF, EGF, PI3K/AKT, *etc.*, and at the same time enhances the migration and invasion capabilities of tumor cells by inducing EMT [18, 19, 37-39]. In addition, TGF-β contributes to the restructuring of the tumor microenvironment by regulating the function of liver cancer-related fibroblasts, thereby supporting the malignant progression of liver cancer. Over the past few years, with an in-depth understanding of the role of TGF-β in HCC, therapeutic strategies targeting TGF-β have gradually become a research focus. Especially in the treatment of advanced liver cancer, the role of TGF-β is regarded as a potential molecular target. Although therapeutic approaches such as immune checkpoint inhibitors have shown significant advancement, the high recurrence rate and drug resistance of HCC remain a huge challenge in clinical treatment. Therefore, exploring more effective TGF-β targeted treatment strategies and revealing the precise mechanism of the involvement of TGF-β in HCC progression remain important directions for future research. This study performed a bibliometric analysis of TGF-β and HCC-related research from the WoSCC database over the past 20 years, highlighting research trends and key findings in the field while offering valuable insights for future studies.

### 4.1 Development Trend Analysis

From 2000 to 2005, the research on TGF-β and HCC remained stable, with an average of about 37 papers published annually. Beginning in 2006, the number of studies has increased dramatically. Although it has declined slightly since then, it is still higher than the level before 2017, indicating that this field has become a research hotspot and continues to have growth potential. This trend may be linked to HCC's emergence as a significant global health issue, especially in some developing countries [40]. As the incidence of HCC increases year by year, the scientific community has gradually deepened its understanding of the disease. TGF-β, a growth factor, has a complex twofold role in the initiation, progression, and metastasis of HCC, and the study of its mechanism of action has attracted the attention of more and more scholars. In addition, the limitations of traditional treatment methods have become apparent, and the study of TGF-β as a potential molecular target in HCC immunotherapy and targeted therapy has become increasingly popular.

### 4.2 Country-level Analysis of Paper Publication Counts

From the perspective of international cooperation and scientific research distribution cooperation network analysis, global research in the field of TGF-β and HCC covers 79 countries, and 43 countries have published 5 or more papers. The cooperation relationship between China and the United States is particularly close, and China's research output is significantly ahead. In addition, research cooperation has a strong international characteristic, but there is no significant clustering trend, suggesting that global research in this field is fairly balanced and broadly distributed. From the perspective of scientific research contribution, China is the largest research contributor in this field, publishing 1,494 papers, occupying an absolute dominant position. The United States follows closely with 553 papers, while countries such as Japan, Germany, and South Korea have relatively few contributions. The reason for this trend may be that China has long been at a high level in the world in terms of the incidence and mortality of liver cancer. As one of China's major public health problems, liver cancer has stimulated Chinese research institutions and scholars to conduct in-depth research in this field [41]. The high incidence of liver cancer has prompted Chinese researchers to focus more on the exploration of related pathological mechanisms, early diagnosis technologies, targeted treatment methods, *etc.* TGF-β, as a crucial factor in HCC progression, has attracted a lot of research attention [42].

### 4.3 Institutional Influence Institutional Analysis

Fudan University, Sun Yat-sen University, Huazhong University of Science and Technology and Shanghai Jiao Tong University have dominated studies in this domain, highlighting Chinese research strength in this area. Fudan University leads in publication count and is also ranked highly for total citations. Although Chinese institutions dominate in terms of publications, the top citations are mainly from the University of Barcelona in Spain and Naval Medical University in China, which also reflects the strong influence of the research of these institutions.

### 4.4 Analysis of Journals and Journal Citations

In the field of TGF-β and HCC, 29 of the 720 journals published 20 or more related papers. The American journal Hepatology led in both publication count and citation numbers, which shows the significance of this journal in the domain of liver disease research. Although Hepatology published the most articles, the journal with the top average citation frequency was the Journal of Hepatology, which indicates that the journal also holds significant academic influence in the research areas of HCC and TGF-β. In addition, through co-citation analysis and Citespace double-graph overlay, this study revealed the close connection and synergy between different disciplines in the research related to TGF-β and HCC. Hepatology, Cancer Research, and Journal of Biological Chemistry, which have a large number of journal citations, show their core position in this field. The different color nodes of the journals reflect the distribution of research in different fields and emphasize the intersection and cooperation between disciplines. The results show that the journal topics are mostly concentrated in the disciplines of oncology, cell biology, and molecular immunology (Figs. **5A** and **B**), indicating that in-depth research on tumor genes, cell mechanisms, and signaling pathways is more conducive to understanding the molecular basis of tumor occurrence and development, and revealing how tumor cells promote tumor growth and metastasis through gene mutations, signal transduction disorders, and interactions with the cell microenvironment. This development trend shows that research hotspots are gradually focusing on the precise cellular mechanism level. Over the last few years, the advancement of single-cell technologies has significantly propelled the depth of cancer research. Techniques such as single-cell RNA sequencing and single-cell ATAC-seq enable researchers to analyze gene expression, epigenetic modifications, and cell-to-cell interactions at the single-cell level [43, 44]. The complex interactions between cancer-associated fibroblasts (CAFs) and other cellular components of the tumor microenvironment (TME) in HCC, particularly among CAFs, tumor cells, and tumor-associated neutrophils (TANs), play a crucial role in cancer progression. CAFs promote the secretion of the chemokine CXCL6 and TGF-β by tumor cells through the release of cardiotrophin-like cytokine factor 1 (CLCF1). These factors not only enhance tumor cell stemness but also facilitate TAN infiltration and polarization. Furthermore, CXCL6 and TGF-β activate the ERK1/2 signaling pathway in CAFs, leading to increased CLCF1 production, thus forming a positive feedback loop that accelerates tumor progression [45]. Moreover, research shows that lactate can impact the role of regulatory T cells (Tregs) within the tumor microenvironment (TME). Lactate promotes the binding between MOESIN and TGF-β receptor I by acetylating the Lys72 site of MOESIN, thereby stabilizing and promoting Treg functionality. The degradation of lactate not only reduces Treg induction but also significantly enhances the anti-tumor immune response and suppresses tumor growth [46].

### 4.5 Author Citation Analysis

Fabregat, Isabel, and Giannelli, Gianluigi are the most influential authors in the field, with 47 and 43 papers published, respectively, and the most citations (3241 and 3239 times). Regarding average citation count rate, Dooley, Steven, and Mishra, Lopa ranked first with 101.19 and 77.72 times, respectively. In addition, the total number of citations of co-cited authors Llovet, JM and Massagué, J remained high, demonstrating the scholarly influence of their team in TGF-β and HCC research.

### 4.6 High-frequency Keyword Analysis

By examining high-frequency keywords in studies on HCC and the TGF-β signaling pathway, it can be found that this field mainly focuses on three key molecular mechanisms: liver fibrosis, tumor metastasis, and cell apoptosis.

Advanced liver fibrosis and cirrhosis are well-established as the major risk factors for HCC. Approximately 90% of HCC cases develop in the context of liver cirrhosis [47]. Cancer-associated fibroblasts (CAFs) are pivotal in the fibrotic microenvironment, influencing the initiation and progression of HCC through various mechanisms [48, 49]. CAFs are mainly differentiated from hepatic stellate cells (HSCs). After activation, these cells will transform into myofibroblasts. Myofibroblasts express and secrete a variety of matrix metalloproteinases (MMPs) and their inhibitors (TIMPs), regulating the degradation and balance of the extracellular matrix (ECM). In the process of liver fibrosis, overexpression of TIMPs inhibits the activity of MMPs, leading to abnormal accumulation of ECM [50-52]. Although CAFs are mainly derived from HSCs, some CAFs may be transformed from other cells, such as parenchymal cells generated through EMT, bone marrow-derived mesenchymal stem cells (MSCs) and fibrocytes, mesothelial cells, and hilar fibroblasts (HFs) [53, 54]. In this process, TGF-β, as a key regulator of fibrosis and tumor microenvironment, runs through the development of HCC. TGF-β activates HSCs and PFs, induces CAFs to produce fine ECM, and promotes the formation of tumor matrix [49, 55, 56]. In addition, TGF-β-driven signaling pathways also enhance the angiogenesis ability of tumor cells by upregulating cytokines and angiogenic factors secreted by CAFs [57]. More importantly, TGF-β can reduce tumor immune surveillance through its action on the immunosuppressive microenvironment, further promoting the immune escape of HCC. In cholestatic liver fibrosis, TGF-β regulates the activation of PFs through interaction with the MUC16-Thy1-TGFβRI complex. CAFs also facilitate EMT *via* the TGF-β signaling pathway, giving tumor cells stronger migration and invasion capabilities [49, 57, 58]. Overall, TGF-β, as an important regulator of CAF function, not only plays a core role in liver fibrosis but also drives HCC initiation and progression through various mechanisms, positioning it as a potential target for HCC treatment.

TGF-β plays a dual function in tumor metastasis, particularly during the advanced stages of cancer [59]. When cancer cells develop resistance to the cell quiescence impact of TGF-β, TGF-β directly promotes tumorigenesis and metastasis by inducing EMT. EMT contributes to embryogenesis and tissue repair during regular physiological processes but may be abnormally activated under pathological conditions, promoting fibrotic diseases and tumorigenesis [59]. The biological characteristics of EMT include reduced E-cadherin expression along with elevated levels of vimentin and N-cadherin, resulting in loss of cell polarity, decreased adhesion ability, and acquisition of invasiveness and migration. Cancer cells associated with a mesenchymal phenotype usually exhibit unfavorable prognosis and demonstrate insensitivity to chemotherapy, while the other role of EMT is mainly related to chemotherapy resistance [60, 61]. Studies have shown that EMT is a multi-step dynamic process with a Partial Epithelial-Mesenchymal Transition (P-EMT), and TGF-β mainly induces epithelial cells to transition to a P-EMT state [62]. In addition, TGF-β can also activate P-EMT in tissue fibrosis, indicating its wide range of effects under different pathological conditions [63].

HCC evades apoptotic signaling through various mechanisms, thereby promoting tumor initiation and progression. HCC cells suppress key apoptotic signaling molecules (such as p53 and Fas), upregulate anti-apoptotic proteins, and activate pro-survival signaling pathways, inhibiting apoptosis *via* both mitochondrial-mediated and death receptor pathways [64-66]. Moreover, TGF-β exhibits dual functionality in HCC: in the early stages, CXXC5 binds to histone deacetylase1 (HDAC1), competing with Smad2/3 for engagement. This eliminates the suppressive impact of HDAC1 on TGF-β signaling, thereby suppressing cell proliferation [67], while in the late stage, TGF-β function is reversed and promotes tumor survival by destroying the positive feedback mechanism related to apoptosis [68]. Since the escape of HCC cells from apoptosis is a key feature of tumor development, therapeutic strategies targeting apoptosis pathways (such as small molecule Bcl-2 inhibitors or Fas agonists) combined with TGF-β inhibitors or immunotherapy may provide new ideas for improving the clinical treatment effect of HCC.

### 4.7 Analyze Research Hotspots Through Keyword Timeline

Analysis of keyword time series evolution graph and keyword burst graph shows that the early stage of this field (2000-2010) mainly focused on the identification of basic mechanisms and receptor proteins, and the research focused on the basic mechanisms related to HCC, especially the function of the TGF-β signaling pathway, the receptor mechanism, and its key part in liver fibrosis and cancer advancement. It is precisely through the detailed exploration of core mechanisms and receptor functions of the TGF-β signaling pathway in the early stage that the theoretical foundation for understanding the tumor microenvironment, the intricate nature of the signaling network, and the development of later therapeutic targets have been laid, providing a direction for in-depth exploration in tumor immune regulation, metabolic regulation, and targeted therapy in the future.

#### 4.7.1 Mid-term Stage (2010-2018)

Research during this period focused on gene expression regulation and the tumor microenvironment. Studies gradually shifted from basic mechanisms to exploring the molecular pathways of HCC, including phenotypic changes and gene regulation involved in tumor progression. Research during this period found that TGF-β promoted the spread of HCC by modulating gene expression and cell phenotypic changes. For example, TGF-β induces EMT in the tumor microenvironment, giving HCC cells stronger invasion and metastasis capabilities. HCC cells often escape TGF-β-mediated inhibition by downregulating CCX5 tumor suppressor gene expression and upregulating oncogene expression [67]. Simultaneously, the regulatory role of the tumor microenvironment has become a research hotspot, including the activation of CAFs, ECM remodeling, and immune escape mechanisms [62, 63, 68]. Drug resistance studies have also pointed out that TGF-β plays a key role in the formation of resistance, providing an important direction for targeted therapy [16].

#### 4.7.2 In the Near Term (2019-2024)

Recent research focuses on the metabolic adjustment of HCC, the immune microenvironment of the tumor, and the potential for targeted therapy. In the near term, research emphasizes the function of TGF-β signaling in metabolic reprogramming and immune regulation. Studies have found that TGF-β can significantly reshape the lipid metabolism of liver cancer cells through EMT. TGF-β first promotes HCC cells to switch from aerobic glycolysis to elevated levels of fatty acid oxidation (FAO) and oxidative phosphorylation (OXPHOS), providing sufficient energy for cell migration and invasion by increasing acetyl-CoA entering the tricarboxylic acid cycle (TCA) [69-71]. At the same time, TGF-β induces upregulation of fatty acid transporters (such as FABPs, SLC27, and CD36), enhancing the uptake of free fatty acids, which is closely related to the EMT phenotype. In addition, TGF-β inhibits lipid synthesis pathways such as fatty acid synthase (FASN), further promoting the switch of cell metabolism to FAO and OXPHOS. HCC cells rely on lipid oxidation during migration and invasion and may restore lipid synthesis to support rapid proliferation after migrating to secondary sites [70, 72, 73]. This metabolic adaptive change reflects energy reprogramming under the regulation of TGF-β, paving a new path for the advancement of precision therapeutic strategies targeting fatty acid oxidation and OXPHOS. Secondly, the regulatory mechanism of the cancer immunological microenvironment and precision immune-based treatment strategies have become research hotspots recently. Investigators have concentrated on how to improve the immunosuppressive environment by targeting key signaling pathways and enhancing the anti-tumor ability of effector immune cells, thereby offering a novel approach to enhance the effectiveness of tumor immunotherapy. Research has demonstrated that TGF-β significantly affects immunosuppression and the formation of the tumor microenvironment in HCC [35]. Liver sinusoidal endothelial cells (LSECs) and HSCs secrete TGF-β, induce the differentiation of regulatory T cells (Treg), upregulate FoxP3 expression through the Smad2/3 pathway, and further enhance Treg secretion of TGF-β, IL-10, and adenosine to inhibit effector T cells (such as CD8^+^ and CTLs), weakening the anti-tumor immune response [35, 74-76]. In addition, TGF-β upregulates the transcriptional expression of PD-1, activates PD-1/PD-L1 signaling, inhibits T cell antigen receptor (TCR) signaling, and leads to T cell exhaustion [77, 78]. At the same time, TGF-β also plays a key role in the transformation of chronic inflammation, liver fibrosis, and cirrhosis to HCC. As an important regulator of inflammatory response, TGF-β can act as an inhibitor of adaptive immunity to reduce excessive inflammatory response and can also promote the secretion of inflammatory cytokines (IL-6, IL-10, *etc.*) in the chronic inflammatory environment to maintain the immunosuppressive state of the tumor microenvironment [79]. In addition, TGF-β effectively triggers EMT, inducing cell phenotype transformation from epithelial to mesenchymal by activating signaling pathways, such as Wnt, Notch, and NF-κB, as well as key EMT inducing factors (such as Snai1/Slug, Twist1/2, and ZEB1/2). This transformation gives cancer cells invasive and stem cell-like properties, which helps them adapt to the inflammatory environment and enhance the malignancy of tumors [79-82]. Based on the immune regulatory function of TGF-β, targeting the TGF-β pathway or blocking the PD-1/CTLA-4 signal is considered to be a potential strategy to enhance the immunotherapy effect of HCC [32, 71]. Combining TGF-β inhibitors with immune checkpoint inhibitors holds promise for enhancing the response rate of HCC individuals to immunotherapy and overcoming the complex immune evasion mechanisms within the tumor microenvironment. This approach offers a more effective solution for precision immunotherapy in HCC.

### 4.8 The Role of Alcohol in HCC Development: Mechanisms and Research Perspectives

With the rapid advancement of molecular biology and immunology, the dose-response relationship and molecular mechanisms of alcohol as a risk factor in HCC are becoming central research topics. Traditionally, alcohol-related HCC was primarily considered to be associated with long-term heavy drinking, especially through the progression of alcoholic liver diseases (such as alcoholic fatty liver, alcoholic hepatitis, and cirrhosis), which can lead to cancer. However, recent studies have gradually revealed that even moderate alcohol consumption may promote HCC development through various mechanisms. This discovery poses new challenges for public health policies and clinical practices [83].

First, the mechanisms of alcohol-induced carcinogenesis: **(1) Epigenetic modifications**: Alcohol and its metabolites (such as acetaldehyde) can interfere with DNA methylation and histone modifications, leading to the silencing of tumor suppressor genes or activation of oncogenes. For example, alcohol may inhibit DNA methyltransferases (DNMTs) or alter histone modification states, affecting the expression of key genes (such as p53 and PTEN), thereby promoting the malignant transformation of hepatocytes [84-86]. **(2) Oxidative stress**: The production of reactive oxygen species (ROS) and reactive nitrogen species (RNS) during alcohol metabolism can lead to DNA damage, lipid peroxidation, and protein dysfunction. Even low-dose alcohol may induce genomic instability and the accumulation of mutations in hepatocytes through cumulative oxidative damage [87, 88]. **(3) Metabolic dysregulation:** Alcohol interferes with liver metabolic homeostasis, affecting glucose, lipid, and amino acid metabolism. Low-dose alcohol may alter the activity of enzymes, such as cytochrome P450 2E1, promoting the production of carcinogenic metabolites while inhibiting the liver’s detoxification function [89, 90].

Second, the primary alcohol metabolites, acetaldehyde, and acetate, play crucial roles in HCC development. Acetaldehyde is a potent DNA crosslinking agent that can directly induce DNA damage and inhibit DNA repair mechanisms. Additionally, acetaldehyde can bind to proteins and lipids, forming adducts that further disrupt cellular function. ROS, by oxidizing DNA bases (*e.g.,* 8-hydroxy-2'-deoxy-guanosine) and damaging mitochondrial function, contribute to the malignant transformation of hepatocytes [91, 92].

Future research must utilize single-cell sequencing technologies to precisely dissect the distribution of alcohol metabolites across different hepatocyte subpopulations and their impact on cellular heterogeneity. Recent advances in single-cell sequencing techniques (such as scRNA-seq and scATAC-seq) have provided unprecedented resolution to reveal the effects of alcohol on hepatocyte heterogeneity. Using single-cell technologies, it is possible to identify specific gene expression profiles and epigenetic changes in hepatocytes exposed to alcohol, thus uncovering potential driver mutations and disrupted signaling pathways. For example, hepatocyte heterogeneity: Alcohol may induce the differentiation or dedifferentiation of hepatocyte subpopulations, promoting the formation of cancer-prone precursors with stem cell-like properties [93, 94]. Single-cell technologies can help identify these precursor cells' characteristic markers, providing insights for early diagnosis. Alcohol exposure may accelerate clonal evolution in hepatocytes, leading to the expansion of dominant clones and the formation of tumors. Single-cell technologies can track the dynamic process of clonal evolution, revealing the time-dependent effects of alcohol in HCC development [95]. Moreover, to fully understand the dose-response relationship between alcohol consumption and HCC risk, more precise models are needed to quantify the impact of different levels of alcohol intake on HCC risk [96]. This includes epidemiological studies: large-scale cohort studies to assess the correlation between low-dose alcohol consumption and HCC incidence, while identifying susceptible populations (*e.g.,* individuals with specific genetic polymorphisms). Experimental models: using organoid models and alcohol-exposure models to simulate liver cell changes under various doses of alcohol, combined with multi-omics data (such as transcriptomics, proteomics, and metabolomics), to systematically analyze alcohol’s carcinogenic mechanisms. A deeper understanding of alcohol's carcinogenic effects at different doses will offer new insights into the prevention and treatment of HCC.

### 4.9 Study Limitations

Despite offering valuable insights into the research landscape of TGF-β and HCC, this study has certain limitations that should be acknowledged. The analysis was confined to publications indexed in the WoSCC, which may have resulted in the omission of relevant studies from other major databases such as PubMed, Scopus, and CNKI. Furthermore, the inclusion of only English-language publications introduces the potential for language bias, possibly overlooking significant findings from studies published in other languages, thereby limiting the comprehensiveness of the analysis.

## CONCLUSION

In summary, this study used software such as CiteSpace, SCImago Graphica, and VOSviewer to conduct a quantitative analysis of the literature related to HCC and TGF-β signaling pathway in the WoSCC database and objectively and impartially visualized the data, systematically displaying the research status of this field from 2000 to 2024. The study encompassed the publication counts across different countries, institutions, and journals. It elaborated on the contributions and connections of various authors in this field. In addition, this study also revealed the temporal evolution trend and hot spot changes in the research of HCC and TGF-β signaling pathways, providing researchers in this field with a clear understanding of the current research situation, which will help promote research progress in this field and lay a solid foundation for future research. Future research should focus on the following key areas: (1) Fine regulation of the TGF-β signaling pathway: A deeper understanding of the mechanisms underlying TGF-β's role in liver cancer is crucial, particularly how its function shifts from tumor-suppressive to tumor-promoting across different stages of the disease. Investigating the cross-regulation between TGF-β and other signaling pathways and how these interactions influence the biological behaviors of HCC cells will provide valuable insights into its contribution to cancer progression. (2) The relationship between alcohol and liver cancer: As the connection between alcohol consumption and liver cancer becomes better understood, future studies should focus on the mechanisms through which alcohol induces epigenetic modifications, oxidative stress, and metabolic disruptions. Specifically, understanding how these effects promote the initiation and progression of hepatocellular carcinoma *via* TGF-β signaling will be critical. Additionally, utilizing single-cell sequencing technologies to analyze the roles of different alcohol metabolites could uncover the potential driving effects of alcohol on the early onset of liver cancer. (3) Tumor immune microenvironment and TGF-β: TGF-β’s critical role in immune evasion makes it a prime target for liver cancer immunotherapy. Future studies should examine the interactions between TGF-β and various immune cells within the tumor microenvironment and explore strategies to restore effector T-cell function by targeting TGF-β signaling. Such approaches could significantly enhance the effectiveness of immunotherapies. Moreover, combining TGF-β inhibitors with immune checkpoint inhibitors may present a promising strategy to improve the therapeutic outcomes in HCC. (4) Metabolic reprogramming and TGF-β: The role of TGF-β in the metabolic reprogramming of HCC is gaining increasing attention. Future research should focus on how TGF-β supports tumor cell migration and invasion by regulating processes such as fatty acid oxidation and aerobic respiration. Identifying the key molecules and targets involved in these processes will provide new avenues for developing precision therapies for liver cancer.

## AUTHORS’ CONTRIBUTIONS

L.-L.Y. conducted data collection, performed the bibliometric analysis, and drafted the initial manuscript. X.C. designed the research methodology, supervised data validation, and contributed to result interpretation. K.-T.H. assisted with literature screening, figure preparation, and manuscript formatting. J.-L.W. conceived and supervised the study, critically revised the manuscript, and approved the final version for submission. All authors have read and approved the final manuscript.

## Figures and Tables

**Table 1:** The leading 10 countries based on the volume of published articles.

**Rank**	**Country**	**Documents**	**Total Citations**	**AAC**
1	China	1494	50324	33.68
2	USA	553	39314	71.09
3	Japan	300	14655	48.85
4	Germany	170	11364	66.85
5	South Korea	154	4917	31.93
6	Italy	153	10772	70.41
7	Spain	109	9008	82.64
8	Egypt	100	2681	26.81
9	France	94	6530	69.47
10	England	81	6457	79.72

**Abbreviation:** AAC: Average Article Citations.

**Table 2:** Top 10 authors in terms of number of articles published.

**Rank**	**Author**	**Country**	**Documents**	**Citations**	**AAC**
1	Fabregat, Isabel	Spain	47	3241	68.96
2	Giannelli, Gianluigi	Italy	43	3239	75.33
3	Bertran, Esther	Spain	24	1141	47.54
4	Dituri, Francesco	Italy	21	1316	62.67
5	Dooley, Steven	Germany	21	2125	101.19
6	Yang, Yan	China	19	409	21.53
7	Fan, Jia	China	18	1138	63.22
8	Mishra, Lopa	USA	18	1399	77.72
9	Matsuzaki, Koichi	Japan	17	1105	65
10	Sancho, Patricia	Spain	15	1098	73.2

**Abbreviation:** AAC: Average Article Citations.

**Table 3:** Top 10 keywords ranked by the number of published articles.

**Rank**	**Keywords**	**Occurrences**
1	Hepatocellular carcinoma	905
2	TGF-β	354
3	Liver fibrosis	166
4	Metastasis	130
5	Apoptosis	124
6	Epithelial-mesenchymal transition	221
7	Cancer	100
8	Fibrosis	88
9	Inflammation	88
10	Hepatic stellate cells	82

## Data Availability

All data generated or analyzed during this study are included in this published article.

## LIST OF ABBREVIATIONS

HCC: Hepatocellular Carcinoma
TGF-β: Transforming Growth Factor-beta
WoSCC: Web of Science Core Collection
EMT: Epithelial-mesenchymal Transition
CAFs: Cancer-associated Fibroblasts
HSCs: Hepatic Stellate Cells
MMPs: Matrix Metalloproteinases
ECM: Extracellular Matrix
MSCs: Marrow-derived Mesenchymal Stem Cells
HFs: Hilar Fibroblasts
P-EMT: Partial Epithelial-Mesenchymal Transition
HDAC1: Histone Deacetylase1
FAO: Fatty Acid Oxidation
OXPHOS: Oxidative Phosphorylation
TCA: Tricarboxylic Acid Cycle
FASN: Fatty Acid Snthase
LSECs: Liver Sinusoidal Endothelial Cells
TCR: T cell Antigen Receptor
DNMTs: DNA Methyltransferases
ROS: Reactive Oxygen Species
RNS: Reactive Nitrogen Species
CNKI: China National Knowledge Infrastructure
