# Supplementary material for: Global Trends in Hepatocellular Carcinoma and TGF-β Research: A Bibliometric and Visualization Analysis from 2000 to 2024
Source: Curr Protein Pept Sci. 2025 Jun 3;27(1):92–110. doi: 10.2174/0113892037378714250529063227 (PMC13223496; doi:10.2174/0113892037378714250529063227)
Supplement: Supplementary file 1 [file CPPS-27-1-92_SD1.pdf]

## Supplementary Materials

# Global Trends in Hepatocellular Carcinoma and TGF-β Research: A Bibliometric and Visualization Analysis from 2000 to 2024

Liu-Lin Yang[1,2], Xing Chen[2,3], Kai-Ting Huang[1,2] and Ji-Long Wang[1,2,*]

[1]*Department of Hepatobiliary Surgery, The First Affiliated Hospital of Guangxi Medical University, Nanning, China;* [2]*Guangxi Key Laboratory of Enhanced Recovery after Surgery for Gastrointestinal Cancer, The First Affiliated Hospital of Guangxi Medical University, Nanning, China;* [3]*Department of Ultrasound, The First Affiliated Hospital of Guangxi Medical University, Nanning, China*

**Table S1 The leading 10 institutions based on the volume of published articles**

| Rank | Organization | Country | Documents | Citations | AAC |
|---|---|---|---|---|---|
| 1 | Fudan University | China | 105 | 4114 | 39.18 |
| 2 | Sun Yat-sen University | China | 84 | 3629 | 43.20 |
| 3 | Huazhong University of Science and Technology | China | 73 | 2478 | 33.95 |
| 4 | Shanghai Jiao Tong University | China | 68 | 3122 | 45.91 |
| 5 | Zhejiang University | China | 67 | 2124 | 31.70 |
| 6 | University of Barcelona | Spain | 53 | 5112 | 96.45 |
| 7 | Anhui Medical University | China | 50 | 1522 | 30.44 |
| 8 | Second Military Medical University | China | 49 | 4652 | 94.94 |
| 9 | Southern Medical University | China | 48 | 1780 | 37.08 |
| 10 | Nanjing Medical University | China | 48 | 1812 | 37.75 |

AAC: average article citations.

1389-2037/26 © 2026 Bentham Science Publishers

**Table S2 Top 10 journals in terms of number of articles published**

| Rank | Journal | Country | IF(2024) | JCR(2024) | Documents | Citations | ACC |
|---|---|---|---|---|---|---|---|
| 1 | Hepatology | USA | 12.9 | Q1 | 106 | 11920 | 112.45 |
| 2 | Oncotarget | USA | 2.54 | Q2 | 67 | 2778 | 41.46 |
| 3 | International Journal of Molecular Sciences | Switzerland | 4.9 | Q1 | 66 | 1803 | 27.32 |
| 4 | PLoS ONE | USA | 2.9 | Q1 | 65 | 2172 | 33.42 |
| 5 | World Journal of Gastroenterology | China | 4.3 | Q1 | 55 | 2027 | 36.85 |
| 6 | Oncogene | England | 6.9 | Q1 | 46 | 4140 | 90 |
| 7 | Cancer Letters | Netherland | 9.1 | Q1 | 42 | 2284 | 54.38 |
| 8 | Cancers | Switzerland | 4.5 | Q1 | 40 | 625 | 15.63 |
| 9 | Scientific Reports | England | 3.8 | Q1 | 38 | 1070 | 28.16 |
| 10 | Journal of Hepatology | Netherland | 26.8 | Q1 | 36 | 4302 | 119.5 |

IF: Impact Factor; JCR: Journal Citation Reports; AAC: Average Article Citations.

**Table S3. Top 10 co-cited journals in terms of number of articles issued**

| Rank | Journal | Country | IF(2024) | JCR(2024) | Citations |
|---|---|---|---|---|---|
| 1 | Hepatology | USA | 12.9 | Q1 | 9059 |
| 2 | Cancer Research | USA | 12.5 | Q1 | 5740 |
| 3 | Journal of Biological Chemistry | USA | 4 | Q2 | 4680 |
| 4 | Journal of Hepatology | Netherland | 26.8 | Q1 | 3936 |
| 5 | Oncogene | England | 6.9 | Q1 | 3793 |
| 6 | Cell | USA | 45.5 | Q1 | 3734 |
| 7 | Gastroenterology | USA | 25.7 | Q1 | 3676 |
| 8 | Proceedings of the National Academy of Sciences of the United States of America | USA | 9.4 | Q1 | 3509 |
| 9 | Nature | England | 50.5 | Q1 | 3358 |
| 10 | PLoS ONE | USA | 2.9 | Q1 | 2847 |

IF: Impact Factor; JCR: Journal Citation Reports.

**Table S4. Top 10 co-cited authors in terms of number of Citations**

| Rank | Co-cited authors | Citations |
|---|---|---|
| 1 | Llovet, JM | 593 |
| 2 | Massagué, J | 513 |
| 3 | Giannelli, G | 500 |
| 4 | Friedman, SL | 450 |
| 5 | Derynck, R | 328 |
| 6 | El-serag, HB | 294 |
| 7 | Thiery, JP | 282 |
| 8 | Matsuzaki, K | 271 |
| 9 | Jemal, A | 270 |
| 10 | Zhang, Y | 242 |

**Table S5. Top 10 co-cited references**

| Rank | Title | Journals | Authors | Year | Citations |
|---|---|---|---|---|---|
| 1 | TGFbeta in Cancer | Cell | Joan Massagué | 2008 | 174 |
| 2 | Liver fibrosis | The Journal of clinical investigation | Ramón Bataller | 2005 | 151 |
| 3 | Global Cancer Statistics | CA: a cancer journal for clinicians | Ahmedin Jemal | 2011 | 151 |
| 4 | Sorafenib in advanced hepatocellular carcinoma | The New England journal of medicine | Josep M Llovet | 2008 | 147 |
| 5 | Epithelial-mesenchymal transitions in development and disease | Cell | Jean Paul Thiery | 2009 | 124 |
| 6 | Hallmarks of cancer: the next generation | Cell | Douglas Hanahan | 2011 | 113 |
| 7 | Hepatocellular carcinoma: epidemiology and molecular carcinogenesis | Gastroenterology | Hashem B El-Serag | 2007 | 111 |
| 8 | TGF-beta signaling in tumor suppression and cancer progression | Nature Genetics | R Derynck | 2001 | 108 |
| 9 | Smad-dependent and Smad-independent pathways in TGF-beta family signalling | Nature | Rik Derynck | 2003 | 108 |
| 10 | Transforming growth factor-beta gene expression signature in mouse hepatocytes predicts clinical outcome in human cancer | Hepatology | Cédric Coulouarn | 2008 | 102 |
